# Loss of IL10 Signaling Promotes TLR9-Driven Liver Dysfunction by Allowing T Cell Receptor-Mediated T Cell Activation

**DOI:** 10.21203/rs.3.rs-10449618/v1

**Published:** 2026-08-05

**Authors:** Grace Fisler, Matthew Eremita, Shivani Chhabra, Mariana R. Brewer, Mei Qi, Joyce Hui Yuen, Clifford S. Deutschman, Matthew D. Taylor

**Affiliations:** Cohen Children’s Medical Center of New York; Cohen Children’s Medical Center of New York; Cohen Children’s Medical Center of New York; Cohen Children’s Medical Center of New York; Cohen Children’s Medical Center of New York; Cohen Children’s Medical Center of New York; Cohen Children’s Medical Center of New York; Cohen Children’s Medical Center of New York

**Keywords:** IL10, macrophage activation syndrome, inflammation, autoimmunity, liver dysfunction

## Abstract

**Objective:**

Deficient IL10 signaling can contribute to macrophage activation syndrome. We hypothesized that in TLR-9-mediated inflammation, blocking IL10 would increase T cell activation and worsen organ dysfunction.

**Methods:**

C57BL/6 mice (n = 3–11/cohort) were injected with A) CpG ODN1826, a TLR9 agonist, 50μg every other day for 5 doses on days − 8 — 0 (CpGLo), B) CpG 500μg on Day 0 (CpGHi), or C) CpGHi+ IL10-R blocking antibody. OTI and OTII mice, which respectively contain CD8 and CD4 cells with transgenic T cell receptors, do not respond to antigens. Wild-type C57Bl/6, OTI, and OTII mice received CpGHi+ IL10-R. Baseline (untreated) mice were included in each experiment. Animals were sacrificed one day post-challenge.

**Results:**

The CpGHi cohort had lower WBC (p < 0.0001), higher ferritin (p = 0.0035) and higher ALT (p < 0.05) and lower bile acid transporter mRNA levels (p < 0.05). The CpGHi cohort had increased percentages of B cells expressing activation markers (CD80, CD86, and MHC-I, p < 0.05), of dendritic cells expressing CD86 (p = 0.03), and of CD4 (p < 0.0001) and CD8 (p = 0.025) T cells expressing CD69. Compared to CpGHi, CpGHi+ IL10-R treatment increased Nur77 expression on CD4 and CD8 T cells (p < 0.0001), did not alter innate immune cells, and was associated with worse liver function (p < 0.05). We noted significant expansion of Nur77^+^CD69^+^ CD8 T cells in OTI and OTII mice, indicating an autoreactive CD8 T cell response.

**Conclusions:**

In TLR9-induced hyperinflammation, IL10 restrained a feedforward loop of innate and T cell activation. The specificity of the innate and T cell interaction is unclear and may represent an autoimmune activation of CD8 T cells.

## Introduction

Macrophage activation syndrome (MAS) is a life-threatening complication of several rheumatologic disease processes. MAS is driven by cytokine release and what would appear to be non-specific immune hyperactivation, resulting in unrelenting fever, pancytopenia, coagulopathy, hepatosplenomegaly, multi-organ dysfunction and mortality[1–7]. MAS mimics a spectrum of immune hyperactivation syndromes including hemophagocytic lymphohistiocytosis and some patients with septic shock[8–14].

Interleukin-10 (IL10) is a cytokine that promotes T cell tolerance and the development of T regulatory cells[15]. These leukocytes recognize self-antigen or foreign antigens that should not provoke significant immune responses. Regulatory T cells develop when naïve T cells interact with antigen presenting cells (APCs), such as dendritic cells, B cells or monocytes, in the presence of the appropriate cytokine milieu and inhibitory co-ligand signaling within the immune synapse of the T cell and APC[16]. Conversely, during severe inflammation, these APCs can promote inflammatory activation of T cells, leading to effector responses. APCs communicate with T cells to modulate the strength of T cell receptor (TCR)-mediated activation[17]. IL10 is essential in modulating these T cell-APC interactions.

Deficient IL10 production and signal transduction, as well as polymorphisms of the IL10 gene family, are potential drivers of pathology in systemic Juvenile Idiopathic Arthritis sJIA, a rheumatologic disorder often complicated by MAS[15, 18, 19]. sJIA patients have a reduced number of IL10-expressing B cells than healthy children[14], yet IL10 gene expression is upregulated in monocytes of patients with active sJIA[20]. In systemic lupus erythematosous (SLE), plasma IL10 levels correlate with more severe disease and are higher in patients who develop MAS compared to those who remain unaffected[14, 19, 21, 22]. Thus, through still unknown mechanisms, disrupted IL10 signaling appears to contribute to the pathogenesis of MAS.

There is emerging evidence of a role for T cells in the pathogenesis of MAS[23–27]. In particular, gene expression pathway analyses in patients with MAS have identified upregulated Toll-like receptor (TLR)-7 and TLR-9 signaling[28]. An MAS-like phenotype can be induced in mice by repeated administration of low-dose CpG DNA (ODN1826), a TLR-9 agonist that is abundant in bacteria[18]. In contrast to clinical MAS and other immune hyperactivation syndromes, low dose CpG-treated mice improve over time[18, 29]. Additionally, the CpGLo MAS model is solely dependent on innate immune activation and does not appear to involve abberent IL10 signaling or T cell responses. Therefore, we set out to investigate the role of IL10 signaling and T cell receptor (TCR)-driven T cell activation in response to TLR activation and the resulting T cell-APC interactions.

We hypothesized that in a model of hyperinflammation, TLR-9 driven innate immunity stimulates T cell receptor activation, which is suppressed by IL10. We hypothesized that loss of IL10 signaling in the presence of TLR-9 agonism would result in worsened morbidity and hepatic and hematologic dysfunction, thereby mechanistically defining a role for IL10 in restraining MAS progression through prevention of TCR activation.

## Materials and Methods

All animal studies were approved by the Institutional Animal Care and Use Committee (IACUC #2022–021) and adhered to National Institutes of Health and Animal Research: Reporting of In Vivo Experiments (ARRIVE) guidelines. C57Bl/6J male mice were obtained from the Jackson Laboratory (Bar Harbor, ME) and maintained in the SPF animal facility on a 12-hour day/night cycle at the Feinstein Institutes for Medical Research. Animals were randomly assigned to treatment groups. Cages were kept in the same facility on the same water and food supplies to minimize confounding. Blinding was not performed as no subjective measures were recorded. Animals were euthanized via cervical dislocation in accordance to ARRIVE guidelines. Anesthesia was not used in this study.

## Exposures

### MAS Models

Four groups of 10-week-old male C57BL/6 mice were studied (n = 3–11/cohort)

1. Baseline (untreated)
2. The classical MAS model: CpG 50μg (ODN1826) injected every other day for 5 doses on days − 8 — 0 (CpGLo)[18].
3. Hyperinflammation: CpG 500μg on day 0 (CpGHi).
4. Hyperinflammation with IL-10 receptor blockade: CpGHi with addition of an IL10-R receptor blocking antibody 1000μg (Clone: 1B1–3A, BioXcell), (CpGHi+ IL10-R).

### Protocol

A cheek bleed was performed on day 1 post-challenge, after which animal were euthanized. Livers were harvested and stored in RNA-later (Thermo Fisher, Bohemia, NY, USA). The spleens were digested and stained for flow cytometry as described below. To determine weight change over time, a cohort of animals were weighed daily to day 6 following challenge.

#### Co-blockade experiments:

Next, co-blockade experiments were performed. Nur77^GFP^ (C57BL/6-Tg(Nr4a1-EGFP/cre)820Khog/J) male and female transgenic mice (n = 4/group) were exposed to CpGHi, CpGHi+ IL10-R, CpGHi+ IL10-R+ MHC-I (Clone: M1/42.3.9.8), or CpGHi+ IL10-R+ MHC-II (Clone: M5/114) and sacrificed 24hrs after exposure following the same procedure as above. The blocking antibodies were administered at a dose of 500ug (BioXcell)[30–32]. In addition to the flow cytometry analysis, these cohorts also underwent imaging flow cytometry to examine immune synapse formation. An Immune Synapse (IS) was defined as an APC (MHCII^+^) in contact with a CD8 T cell (CD8^+^) with polarization of actin at the cell-cell interface, indicating a mature IS. T cell-APC Immune synapses data was obtained on Amnis ImageStream and analyzed using IDEAS 6.2.

### Auto-Reactivity Assessment

OTI (C57BL/6-Tg(TcraTcrb)1100Mjb/J), OTII (B6.Cg-Tg(TcraTcrb)425Cbn/J), and wild-type C57Bl/6 mice (n = 3–4/group) were treated with CpGHi+ IL10-R, euthanized day 1 post-challenge along with a cohort of baseline animals (n = 3–4/cohort). OTI mice express a TCR specific to the Ova_257−264_ peptide in CD8 T cells, while OTII mice express a TCR specific to the Ova_323−339_ peptide in CD4 T cells. These OT TCRs suppress intrinsic TCR expression; therefore, the only T cell response in the transgenic T cells is to ovalbumin protein peptides, which are not naturally present in the transgenic mice[33, 34].

### Hematologic and Hepatic Function Assessments

Peripheral blood was used for complete blood count (Advia 2120i Hematology System, Siemens, Munich, Germany). Serum was collected for ferritin (Thermo Fisher, Bohemia, NY, USA) and alanine aminotransferase (ALT) (ABCAM, Waltham, MA, USA) ELISAs. Spleens were weighed upon sacrifice. Liver homogenate underwent RT-PCR for bile acid transporter transcription (ABCC2, Slc10A1, and Slc1a1). Suppression of these transcription factors indicates hepatic dysfunction[35].

### Characterization of Immune Cell Subpopulations and Plasma Cytokine Concentrations

Following splenic digestion, single cell suspension was obtained by passing spleens through a 70um strainer. Cells were stained with LIVE/DEAD fixable viability dye (Life Technologies, Carlsbad CA); samples stained with either a 14-antibody panel for either T cell markers or for innate and B cell markers. Antibodies to intracellular proteins (FoxP3, IFNγ, IL10, and Nur77) were applied after cells were fixed and permeabilized. A GFP negative littermate control was included in experiments that used Nur77^GFP^ transgenic mice to define GFP positivity. Flow cytometry was performed on a 14-color Ze5 flow analyzer (Bio-Rad). A minimum of 1×10^5^ events were analyzed per sample. If this number of events was not reached, the sample was excluded. Results were analyzed using FlowJo software v10.1 (BD Bioscience, San Jose, CA).

Levels of cytokines from plasma collected and stored at −80°C, were determined using multiplex ELISA (Eve Technologies, Calgary, Alberta, Canada).

### Statistical Analysis

Data from individual animals was compared. It was assumed that three animals per group was sufficient to detect differences. When experiments were repeated data was pooled, thus increasing the sample size for certain comparisons. Categorical data were expressed as frequency (percent) and continuous data as mean (standard deviation) or median (interquartile range). Values of continuous variables that were outside the range of the test were assigned the upper or lower limit of the assay.

Analyses were performed using GraphPad PRISM 9. Clinical and immunologic variables with normally distributed variances (as determined using the D’Agostino-Pearson omnibus test) were compared using one-way ANOVA with Dunnett’s correction for multiple comparisons to the CpGHi cohort with p < 0.05 indicating significance. Some variables required natural logarithmic-transformation and were re-analyzed using ANOVA. Two-way ANOVA with variables of cohort and time was used to assess differences in weight loss over time between cohorts.

For analysis of co-blockade experiments, One-way ANOVA was used to compare subpopulations between baseline, CpGHi, CpGHi+ IL10-R, CpGHi+ IL10-R+ MHC-I, and CpGHi+ IL10-R+ MHC-II treated animals; multiple comparisons with Dunnett’s correction were made comparing each cohort to CpGHi+ IL10-R. Immune synapse data was compared between baseline, CpGHi, and CpGHi+ IL10-R via One-way ANOVA with Tukey’s multiple comparisons test to examine individual differences between all cohorts. Experiments comparing baseline, OTI+CpGHi+ IL10-R, OTII+CpGHi+ IL10-R, and WT+CpGHi+ IL10-R mice used One-way ANOVA with Tukey’s multiple comparisons test to examine individual differences between all cohorts.

## Results

### Hemtaologic and Liver Function and Morbidity (Fig. 1)

MAS is characterized by suppression of peripheral white blood cell counts, elevated ferritin, hepatosplenomegaly, and hepatic dysfunction[1, 4, 5]. Ferritin, a classical biomarker of MAS[3], was higher in the CpGHi cohort than in baseline (p = 0.003) and CpGLo (p = 0.002) mice; ferritin elevation was exaggerated in the CpGHi+ IL10-R cohort. WBC counts were lower in CpGHi compared to baseline (p = 0.0006) and CpGLo (p = 0.005) mice and the absolute lymphocyte count was lower in CpGHi compared to baseline (p = 0.0006) and CpGLo (p = 0.006) mice. IL10-R blockade had minimal effect on these hematologic outcomes.

ALT levels, a marker of stressed hepatocyte function, was also different between the four studied cohorts (p = 0.05); statistical differences in ALT were driven by reduced levels in CpGLo and higher levels in CpGHi+ IL10-R. Transcription of the bile acid transporters ABCC2, Slc1A1, and Slc10A1 reflects the functional ability of hepatocytes; suppression of transcription can be a marker of liver dysfunction[35]. Compared to CpGHi mice, mRNA levels of ABCC2 (p = 0.038) and SCL1a1 (p = 0.007) was suppressed in the CpGHi+ IL10-R cohort. Slc10A1 was different between the 4 studied cohorts (p = 0.002), similarly driven by suppression of transcription in CpGHi+ IL10-R. Finally, CpGHi+ IL10-R suffered more severe weight loss compared to animals exposed to CpGHi alone (p < 0.0001).

### Circulating Cytokine Response (Fig. 2)

Serum cytokines from baseline, CpGLo, and CpGHi+ IL10-R cohorts were compared to CpGHi animals. CpGHi mice compared to baseline and/or CpGLo cohorts had higher levels of G-CSF, IFNγ, KC, IL-6, IL-10, IL-12p70, IP-10, MIP-1β, MIG, RANTES and TNF. All of these levels were higher in CpGHi+ IL10-R mice than in CpGHi mice. M-CSF, which drives macrophage activation, was also elevated CpGHi+ IL10-R compared to CpGHi. These data indicate a circulating cytokine profile associated with hyperinflammation in the animals exposed to CpGHi compared to baseline and CpGLo, which was more exaggerated with loss of IL10 signaling.

### Innate Immune Response (Fig. 3)

While there were no differences in the number of splenic B (CD19^+^/B220^+^) or dendritic cells (CD11c^+^/MHCII^+^, cDCs) noted between cohorts, there were significant changes in expression of activation markers on B cells and cDCs in mice treated with CpGHi compared to baseline and CpGLo. Compared to baseline, CpGHi treated mice had higher percentages of B cells expressing high levels of CD80 (p = 0.039), CD86 (p = 0.01), MHC-I (p = 0.005), and MHC-II (p = 0.01) (data not shown). Further, when overall expression on CpGHi was examined, the Median Fluorescence Intensity (MFI) of CD86 (p = 0.002) and MHCII (p = 0.005) on B cells was higher than both baseline and CpGLo and the expression of MHCI (p = 0.01) was higher than baseline. Similarly, compared to CpGLo, mice treated with CpGHi had higher percentages of cDCs expressing CD80 (p = 0.0003), CD86 (p < 0.0001), and MHC-I (p = 0.0004) (data not shown). When the overall expression of these ligands on CpGHi was examined, the MFI of MHCI on cDCs was higher than both baseline (p = 0.005) and CpGLo (p = 0.0008) and the expression of CD80 (p = 0.007) was higher than CpGLo. There were no notable differences between the CpGHi and CpGHi+ IL10-R cohorts in expression of activation markers on B cells and cDCs.

### Adaptive Immune Response (Fig. 4)

There were no differences in total number of splenic CD4 or CD8 T cells between the four studied cohorts. Compared to baseline and CpGLo, CpGHi animals had a higher percentage of both CD4 and CD8 T cells expressing CD69, a marker of general T cell activation[36]. Similarly, compared to CpGHi, loss of IL10 signaling with CpG activation in the CpGHi+ IL10-R cohort led to a higher percentage of CD4 (p < 0.0001) and CD8 (p < 0.0001) T cells expressing CD69. The proportion of CD4 and CD8 T cells expressing Nur77, a marker of direct TCR activation previously used to identify disease specific T cells in rheumatoid arthritis[37–39], was not different between the studied cohorts. In contrast, compared to CpGHi, the level of Nur77 expression by MFI was significantly higher in both CD4 (p < 0.0001) and CD8 (p < 0.0001) T cells in the CpGHi+ IL10-R cohort. Further, when the proportion of TCR-activated effector T cells, as indicated by dual expression of CD69 and Nur77, was compared to the CpGHi cohort, the CpGHi+ IL10-R cohort had higher percentages of CD69^+^Nur77^+^ TCR activated CD4 (p = 0.002) and CD8 (p = 0.003) T cells. This associated with a significantly higher number of the CD4^+^Nur77^+^CD69^+^ splenic T cell population in CpGHi+ IL10-R compared to CpGHi (p = 0.01), and a significant difference in the CD8^+^Nur77^+^CD69^+^ splenic T cell population driven by higher numbers in the CpGHi+ IL10-R cohort (p = 0.005) (Fig. 4A). Hepatic T cell subpopulations between the four study cohorts were also examined, with similar results (Supplemental Fig. 1).

TCR-activated T cells in response to CpG could represent activation in response to self-antigen. These T cells may act as a tolerogenic checkpoint, preventing further activation. To examine the behavior of T cells activated via T cell receptor (TCR) activation, the regulatory and activated populations of splenic CD4^+^Nur77^+^ and CD8^+^Nur77^+^ T cells were examined[38]. CD8^+^Nur77^+^ T cells were predominantly activated (CD69^+^), nonregulatory (FoxP3^−^) cells in the cohorts exposed to CpGHi, which was exaggerated by the addition of IL10 blockade (p = 0.025). Compared to baseline, both CpGHi (p = 0.007) and CpGHi+ IL10-R (p = 0.0002) animals had a higher percentage of CD8 cells co-expressing CD69 and Nur77.

These data suggest CD4 and CD8 T cell activation is present after exposure to CpGHi compared to baseline and CpGLo, and that this activation is enhanced by the loss of IL10 signaling. Further, in response to CpGHi, CD8 T cells activated via T cell receptors are of the nonregulating, activated effector phenotype.

### Effects of Co-Blockade of IL10 and MHC-I or MHC-II (Fig. 5)

TCR activation occurs in response to immune synapse formation when a T cell interacts with an antigen presenting cell (APC), leading to ligation of the TCR by MHCI for CD8 T cells and MHCII for CD4 T cells. Both B cells and cDCs can act as APCs, guiding T cell responses to inflammation[40]. If T cell activation is occurring in response to TCR signals from APCs, blockade of MHCI or MHCII should prevent TCR activation in response to CpGHi+ IL10-R.

Compared to baseline, mice treated with CpGHi+ IL10-R again demonstrated higher percentages of CD4 and CD8 T cells expressing CD69 or Nur77. Co-expression of CD69 and Nur77 on CD4 T cells was affected by the addition of MHC-blockade: CpGHi+ IL10-R animals had higher percentage of CD4 T cells expressing Nur77^+^CD69^+^ than baseline (p < 0.0001); mice exposed to IL10-R and MHC-II co-blockade had lower percentage of CD4 T cells expressing Nur77^+^CD69^+^ than CpGHi+ IL10-R animals (p = 0.038). This suggests that CD4 T cell TCR activation in the presence of IL10-R blockade is MHC-II-dependent. In contrast, CpGHi+ IL10-R animals again had a higher percentage of CD8 T cells expressing Nur77^+^CD69^+^ than baseline (p < 0.0036). Mice exposed to CpGHi and IL10-R and MHC-I co-blockade did not demonstrate a lower percentage of CD8 T cells expressing Nur77^+^CD69^+^ compared to CpGHi animals (p = 0.80), but did have a lower number of CD8 T cells expressing Nur77^+^CD69^+^ (p = 0.046), again indicating that CD8 T cell TCR activation in the presence of IL10-R blockade is MHC-I-dependent (Fig. 5A).

When immune synapse (IS) analysis of CD8^+^Nur77^+^ T cells was pursued using imaging flow cytometry, the percent of CD8 T cells synapsed with APCs in response to CpGHi was significantly lower than baseline (p = 0.0495) (Fig. 5B). Loss of IL10-R signaling led to recovery of this interaction. Nearly all TCR-activated CD8 T cells were actively engaged in an immune synapse with an APC; the level of MHCI on the APC was not altered by CpGHi or IL10-R blockade. In contrast, the MFI of CD11a and actin, which are involved in immune synapse maturation, at the IS were significantly higher in CpGHi compared to baseline (p = 0.003 and p = 0.01, respectively) and CpGHi+ IL10-R (p = 0.002 and p = 0.01, respectively) animals. These data may indicate that CpGHi leads to significantly stronger immune synapsation, but IL10 prevents elimination of the immune synapsed pair, thereby preventing further immune activation and inflammation.

B) Differences in immune synapse (IS) strength and percentage binding in CD8 + Nur77+ cells between baseline, CpGHi, and CpGHi+ IL10-R by One-Way ANOVA with Tukey’s correction for multiple comparisons to CpGHi. N = 4 animals per cohort. Dots are individual animals, bars are mean, lines are 95% CI. *=p < 0.05, **=p < 0.01; ***=p < 0.001; ****=p < 0.0001.

### Antigen Dependence of T Cell Responses in CpG with Blockade of IL10 (Fig. 6)

Both CD4 and CD8 T cells must undergo positive selection during development with some degree of recognition of self-antigen to remain within the T cell repertoire[41]. While both CD4 and CD8 T cells demonstrated TCR activation with CpGHi, which was enhanced by IL10-R blockade, CD8 T cells have been shown to undergo “antigen non-specific” activation in inflammatory environments. It is unclear if this activation is due to low affinity TCR-MHC interactions or truly cytokine driven and antigen independent. To investigate the requirement for antigen in response to CpGHi+ IL10-R, OTI and OTII mice were treated with CpGHi+ IL10-R (OTI and OTII) and compared to baseline (untreated) animals and wild-type C57Bl/6 mice treated with CpGHi+ IL10-R (WT). As described above, the only T cell response in the transgenic T cells is to ova peptide, which is not naturally present in the transgenic mice. Thus, any observed T cell activation would lack antigen-specificity and thus suggest autoreactivity.

In response to CpGHi+ IL10-R, Nur77^+^CD69^+^ CD4 T cells were significantly expanded in OTI mice compared to baseline (p < 0.0001) and WT mice treated with CpGHi+ IL10-R (p < 0.0001). Conversely, Nur77^+^CD69^+^ CD4 T cells in OTII mice was only slightly higher than baseline and similar to WT animals treated with CpGHi+ IL10-R. In contrast, we found significant expansion of the Nur77^+^CD69^+^ CD8 T cell population in both OTI and OTII mice compared to both baseline and WT animals, indicating an antigen non-specific response by CD8 T cells. OTI mice also had a higher percentage of Nur77^+^CD69^+^ expression in CD8 T cells than observed in WT mice. Similar percentages of CD69^+^Nur77^−^ expression in CD4 and CD8 T cells were observed in OTI and OTII mice, which were higher than baseline animals.

Congruent with exaggerated antigen non-specific T cell activation, both CD4 and CD8 T cells in OTI mice produced more IFNγ than observed in baseline or WT mice. OTII mice did not demonstrate exaggerated IFNγ production by either CD4 or CD8 T cells, suggesting a requirement for CD4 T cell antigen recognition in producing the full immunologic phenotype observed in response to CpGHi+ IL10-R. Finally, while the TCR activated CD4 T cells in OTI mice were partially T regulatory (FoxP3^+^) cells, the overall activation of these T cells still favored effector activation, indicating a significant inflammatory response.

## Discussion

We demonstrated that in our model of TLR9-driven activation, there is TCR activation driven by innate immune cell hyperactivation, which is restrained by IL10 impacting T cell-mediated immune synapse stability. The loss of this IL-10 mediated immune checkpoint causes a cytokine storm and hepatic dysfunction like that observed in MAS. The role of insufficient IL10 signaling in MAS pathogenesis has been previously suggested[18], and literature would support loss of IL10 leading to a loss of T cell regulation[42–45]. Indeed, we found that when IL10 blockade was co-administered with high dose TLR9 agonism, many plasma cytokines associated with hyperinflammation, including IFNγ, IL-6, RANTES, and TNF were higher than in the presence of high dose TLR9 agonism alone.

While TLR9 activation leads to high levels of B cell and dendritic cell activation, this is not significantly affected by IL10. Conversely, T cell receptor-mediated T cell activation was partially suppressed by IL10 signaling. Compared to animals exposed to high dose TLR9 agonism alone, those exposed to high dose TLR9 agonism with concurrent IL10 blockade had higher ferritin, more liver dysfunction, and more weight loss over time. These findings suggest that in our model of TLR9-driven hyperinflammation, IL10 is vital in restraining T cell-mediated immune activation and protecting against liver dysfunction.

As TLR9 driven inflammation is a sterile system, no foreign antigen is introduced to drive T cell-APC interactions. Thus, a component of the hyperinflammation seen may be driven by autoimmune T cell responses. We demonstrated that interruption of T cell-APC interactions through blockade of the MHCI or MHCII complexes partially ameliorates TCR activation and that high levels of TCR activation associated with increased immune synapsation. The reduction in immune synapsation with IL10-R blockade may indicate disruption of the normal inhibition of T cell activation that would occur when the TCR-peptide-MHC complex is weak. A mature T cell-APC synapsation event would lead to elimination of the APC and full activation of the T cell. IL10 seems to prevent full synapse maturation and prevent T cell effector functions that could eliminate the APC driving the inflammatory response. The signal strength of the TCR may be modulated by TLR-driven upregulation of co-ligands (CD80, CD86) or of the MHC itself. Further investigation into the myriad factors involved in regulating T Cell-APC interactions in severe inflammation is warranted.

Our data suggest that in this model of hyperinflammation, IL10 restrains a feedforward loop of innate and T cell activation. The specificity of these T cell-APC interactions is not clear and may represent an autoimmune activation of CD8 T cells. This is supported by the apparent lack of need for specific peptide-binding for CD8 T cells, as observed in our experiments with the OTI and OTII mice. CD4 T cell antigen recognition is similarly unclear but may represent a more immunoregulatory response to self-antigens. The observed activation in both CD4 and CD8 T cells may be due to a loss of specificity[46]. Previous reports have also demonstrated “cytokine-driven” CD8 T cell activation, though these studies did not consider weak interactions as a stimulus for CD8 responses[47].

MAS and other secondary HLH syndromes are characterized by an expansion of CD8 T cells[48]. Our data expands on the knowledge that CD8 T cells are a driver in MAS and HLH pathogenesis by characterizing how CD8 T cells are sensitive to IL10. When exposed to TLR9 agonism with IL10 blockade, CD8 T cells vastly increase their expression of the key activation markers CD69 and Nur77 and their co-expression of CD69 and Nur77. As the TLR9 agonism with IL10 blockade cohort also experienced worsened morbidity and organ dysfunction, lack of IL10 signaling may quell CD8 T cell activation and disease activity.

Our new model of MAS involves administration of one high dose of CpG DNA, a TLR9 agonist. This model may more appropriately model severe life-threatening MAS[18] by consistently inducing hepatic and hematologic dysfunction, high levels of plasma cytokines, and B cell, dendritic cell, and CD4 and CD8 T cell activation. The addition of IL10 blockade exacerbates the hepatic dysfunction and the immune activation seen in clinical MAS[1, 2], further supporting that a loss of IL10 signature in patients at risk for MAS can promote development of the disease. This knowledge extends beyond MAS and HLH as patients with other systemic autoimmune diseases and sepsis, a syndrome with major worldwide impact, may have impaired T cell IL10 production[49]. Interestingly, unlike hepatic dysfunction, hematologic dysfunction did not worsen with IL10-R blockade. This suggests disparate immunologic pathways leading to dysfunction in these two systems.

This study has both limitations and strengths. The use of high dose CpG to induce a model of hyperinflammation is artificial and may not represent the full immune response seen in patients. Plasma cytokine levels may not represent true activity as cytokine signaling is typically paracrine, however documentation of the splenic immune response and immune synapse visualization allowed for a more complete picture of immune activity. Further work exploring the role of IL10 in both preclinical models and human disease may be pursued in many areas of medical research.

Our work suggests several important new directions in the study of T cell responses to severe inflammation. The role of this inhibitory system in other hyperinflammatory syndromes, including in HLH and sepsis is warranted. Further, the role of T cell activation and T cell-APC interactions in human patients may represent an area of significant research that could identify sJIA or other rheumatologic patients either at risk for MAS or for further progression of disease. Finally, analysis of components of the immune synapse could identify areas that may be ripe for future targeting for immunomodulation.

In a model of inflammation induced by high dose TLR9 agonism administration, IL10 was vital in preventing both activation of the innate and adaptive immune systems and in attenuating disease activity. Further work to elucidate the role of IL10 in diseases of inflammation may gain important insights into disease pathogenesis.
